# Supplementary material for: Observation of High Magnetic Bistability in Lanthanide (Ln = Gd, Tb and Dy)-Grafted Carbon Nanotube Hybrid Molecular System
Source: Int J Mol Sci. 2023 Aug 1;24(15):12303. doi: 10.3390/ijms241512303 (PMC10418393; doi:10.3390/ijms241512303)
Supplement: Supplementary file 1 [file ijms-24-12303-s001.zip › ijms-2519938-supplementary.pdf]

Observation of magnetic bistability in Lanthanide (Ln = Gd, Dy, Tb) grafted carbon nanotube hybrid molecular system

V.R. Sodisetti[1], Andreas Lemmerer[2], D. Wamwangi[3], S. Bhattacharyya[1*]

[1]Nano-Scale Transport Physics Laboratory, School of Physics, University of the Witwatersrand, Johannesburg Wits 2050, South Africa. svr.quantum@gmail.com
[2]Molecular Sciences Institute, School of Chemistry, University of the Witwatersrand, Johannesburg 2050, South Africa. Andreas.Lemmerer@wits.ac.za
[3]DSI-NRF Centre of Excellence in Strong Materials and School of Physics, University of the Witwatersrand, Johannesburg Wits 2050, South Africa. Daniel.Wamwangi@wits.ac.za
[*]Correspondence: Somnath.Bhattacharyya@wits.ac.za

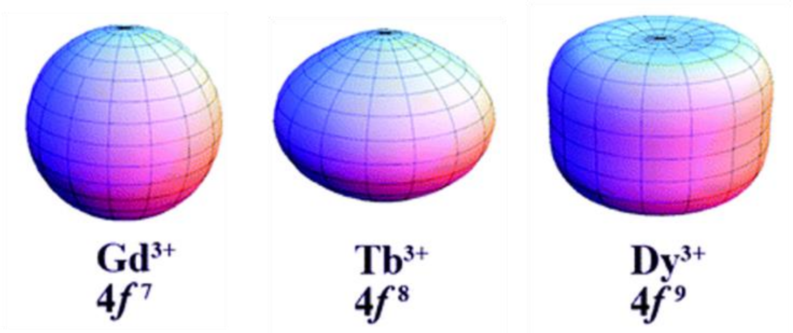

Figure S1. Perceptive view of Gd, Tb, and Dy electronic systems.

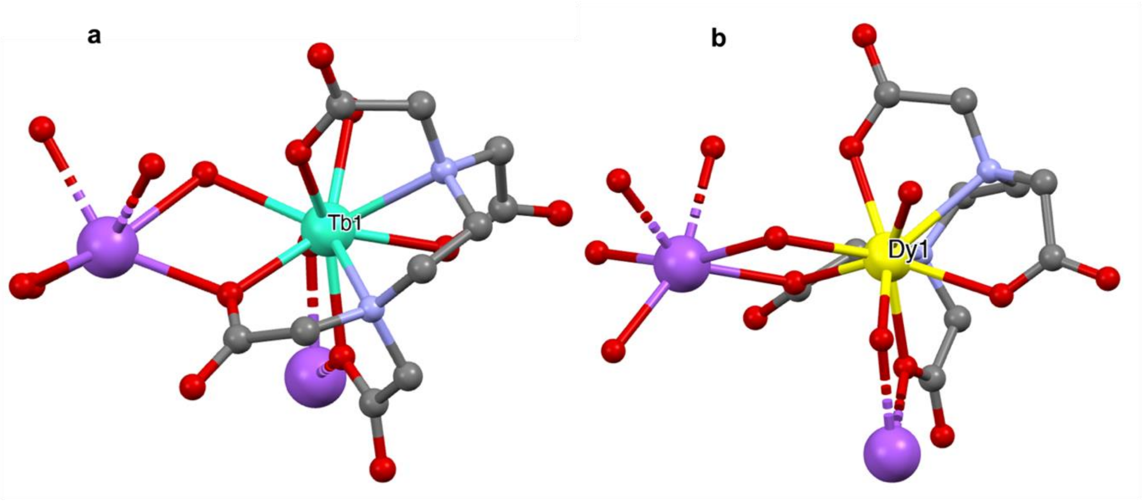

Figure S2. Single crystal X-ray structure of a) Tb EDTA and b) Dy EDTA molecular unit. Hydrogen and water molecules are removed for better presentation.

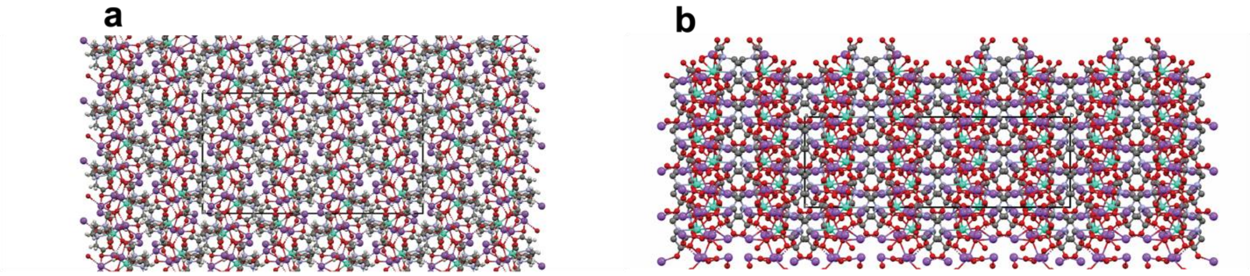

Figure S3: Packing diagram viewed down the *c*-axis. a) Tb-EDTA. b) Dy-EDTA.

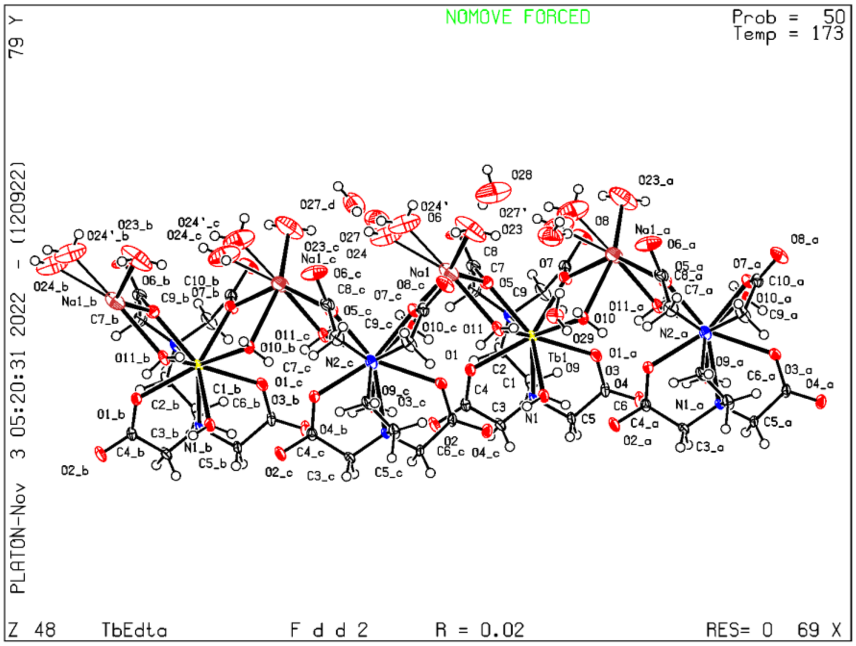

Datablock: TbEdta

Bond precision: C-C = 0.0072 A Wavelength=0.71073

Cell:	a=35.306(3)	b=19.2934(15)	c=12.0542(10)
	alpha=90	beta=90	gamma=90
Temperature:	173 K		

	Calculated	Reported
Volume	8211.0(12)	8211.0(12)
Space group	F d d 2	F d d 2
Hall group	F 2 -2d	F 2 -2d
Moiety formula	C10 H22 N2 Na O13 Tb, 1.5(H O0.50), 2(H2 O)	C10 H22 N2 Na O13 Tb, 2(H O0.50), 2(H2 O)
Sum formula	C10 H28 N2 Na O16 Tb	C10 H28 N2 Na O16 Tb
Mr	614.26	614.25
Dx,g cm-3	1.988	1.988
Z	16	16
Mu (mm-1)	3.548	3.548
F000	4896.0	4896.0
F000’	4896.66	
h,k,lmax	46,25,15	46,25,15
Nref	4943[2588]	4919
Tmin,Tmax	0.136,0.178	0.655,0.747
Tmin’	0.077	

Correction method= # Reported T Limits: Tmin=0.655 Tmax=0.747
AbsCorr = MULTI-SCAN

Data completeness= 1.90/1.00 Theta(max)= 27.998

R(reflections)= 0.0166(4919) wR2(reflections)= 0.0436(4919)

S = 1.491 Npar= 282

Figure S4. Single crystal structure of Tb-EDTA molecule.

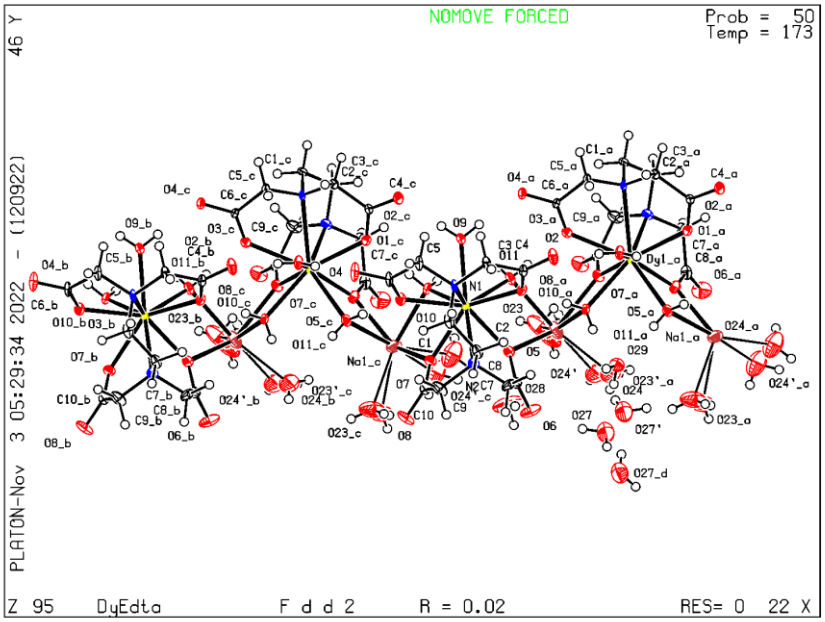

Datablock: DyEdta

```
Bond precision:   C-C = 0.0098 A            Wavelength=0.71073

Cell:              a=19.3107(18)     b=35.216(3)     c=12.0558(11)
                   alpha=90          beta=90         gamma=90
Temperature:       173 K

                  Calculated                  Reported
Volume            8198.5(13)                  8198.6(13)
Space group       F d d 2                     F d d 2
Hall group        F 2 -2d                     F 2 -2d
Moiety formula    C10 H16 Dy N2 Na4 O13       C10 H16 Dy N2 Na4 O13
Sum formula       C10 H16 Dy N2 Na4 O13       C10 H16 Dy N2 Na4 O13
Mr                626.71                      626.71
Dx,g cm-3         2.031                       2.031
Z                 16                          16
Mu (mm-1)         3.796                       3.796
F000              4864.0                      4864.0
F000'             4865.34
h,k,lmax          27,49,16                    27,49,16
Nref              5965[ 3115]                 5946
Tmin,Tmax         0.182,0.490                 0.596,0.746
Tmin'             0.114

Correction method= # Reported T Limits: Tmin=0.596 Tmax=0.746
AbsCorr = MULTI-SCAN

Data completeness= 1.91/1.00        Theta(max)= 29.996

                                                   wR2(reflections)=
R(reflections)= 0.0261( 5933)                      0.0677( 5946)

S = 1.305                 Npar= 274
```

Figure S5. Single crystal structure of Dy-EDTA molecule.
